# Endogenous H_2_O_2_-Sensitive and Weak Acidic pH-Triggered Nitrogen-Doped Graphene Nanoparticles (N-GNMs) in the Tumor Microenvironment Serve as Peroxidase-Mimicking Nanozymes for Tumor-Specific Treatment

**DOI:** 10.3390/ma14081933

**Published:** 2021-04-13

**Authors:** Danyang Liang, Yongzhen Yang, Gongjian Li, Qin Wang, Heting Chen, Xiaoyuan Deng

**Affiliations:** 1 MOE Key Laboratory of Laser Life Science & Institute of Laser Life Science, College of Biophotonics, South China Normal University, Guangzhou 510631, China; liangdanyang12@163.com (D.L.); purestarszz@163.com (Y.Y.); m15875784450@163.com (G.L.); wangqinyier@163.com (Q.W.); chen_he_ting@163.com (H.C.); 2 Guangdong Provincial Key Laboratory of Laser Life Science, College of Biophotonics, South China Normal University, Guangzhou 510631, China; 3 Guangzhou Key Laboratory of Spectral Analysis and Functional Probes, College of Biophotonics, South China Normal University, Guangzhou 510631, China

**Keywords:** POD-mimicking nanozyme, N-doped graphene, high catalytic activity, pH-triggered, tumor-specific treatment, tumor microenvironment

## Abstract

Nanozymes are emerging as a promising strategy for the treatment of tumors. Herein, to cope with the tumor microenvironment (TME), weak acidity (pH 5.6 to 6.8) and trace amounts of overexpressed hydrogen peroxide (H_2_O_2_) (100 µM–1 mM), we report nitrogen-doped graphene nanomaterials (N-GNMs), which act as highly efficient catalytic peroxidase (POD)-mimicking nanozymes in the TME for tumor-specific treatment. N-GNMs exhibit POD catalytic properties triggered by a weakly acidic TME and convert H_2_O_2_ into highly toxic hydroxyl radicals (•OH) thus causing the death of tumor cells while in the neutral pH surroundings of normal tissues, such catalysis is restrained and leaves normal cells undamaged thereby achieving a tumor-specific treatment. N-GNMs also display a high catalytic activity and can respond to the trace endogenous H_2_O_2_ in the TME resulting in a high efficiency of tumor therapy. Our in vitro chemical and cell experiments illustrated the POD-like activity of N-GNMs and in vivo tumor model experiments confirmed the significant inhibitory effect of N-GNMs on tumor growth.

## 1. Introduction

Tumor treatment faces essential challenges in tumor-specific and high efficiency treatment [1]. Nanozymes, the catalytic nanoparticles with enzyme-like properties that can catalyze enzyme reactions with comparable activity and robust stability, have recently attracted great interest in tumor treatment [2,3]. Tumor tissue forms a specific tumor microenvironment (TME) exhibiting hallmarks of mild acidity and an overexpression of hydrogen peroxide (H_2_O_2_) [4]. These peculiar features in the TME can thus be exploited as a strategy for tumor-specific treatment by introducing a peroxidase (POD)-mimicking nanozyme [5]. In the presence of a POD-mimicking nanozyme, H_2_O_2_ can be decomposed into hydroxyl radicals (•OH) under acidic conditions [6]. The produced •OH is a highly toxic substance that can attack and oxidize most organic molecules (cell proteins, cell proteins, etc.) at a rate constant of up to 10^6^–10^9^ M·s^−1^, thereby leading to apoptosis or the necrosis of tumor cells [7,8,9]. POD-mimicking nanozymes have been greatly developed. Nevertheless, POD-mimicking nanozymes adapted to a specific TME for highly efficient tumor-specific treatment are still required.

Due to glycolysis when the glucose oxidation capacity is not enough to maintain the energy for tumor cell proliferation, tumor cells create an acidic TME. In normal cells, glycolysis is inhibited and the pH is controlled at approximately 7.4 [10,11]. The catalytic performance of POD-mimicking nanozymes is acidic pH-triggered [6]. POD-like catalytic activities are activated in the acidic TME to generate highly toxic •OH and cause tumor cell death while this does not occur in the neutral pH environment of normal tissues, leaving the normal cells unharmed. The acidic TME hence becomes a trigger for tumor-specific treatment. Unfortunately, the TME merely presents weak acidity (pH 5.6 to 6.8). Most current POD-mimicking nanozymes are only sensitive to low pH acidic conditions and can only respond to intracellular endo/lysosomes (pH 4.5 to 5.5) in tumor treatment [12,13,14]. POD-mimicking nanozymes with a weakly acidic TME response are thus highly desired.

Induced by the disproportionation of superoxide dismutase in mitochondria, H_2_O_2_ in the TME is overproduced compared with that in normal tissues but endogenous H_2_O_2_ in the TME is still at the microscale of 100 µM–1 mM [15,16]. For those POD-mimicking nanozymes utilized in organisms, in cases of low POD-like catalytic activity, endogenous H_2_O_2_ in the TME is insufficient. Therefore, catalase inhibitors [17], glucose oxidase [18] or exogenous H_2_O_2_ [19] are directly added to increase the concentration of H_2_O_2_. Alternatively, certain synergistic treatments such as external energy fields (heat, [20] microwave [21] or near infrared (NIR) light [22] fields) are exerted or other functional materials are composited to increase the therapeutic performance. For example, highly catalytically active metal particles (Fe [23], Cu [24] nanoparticles) are incorporated for synergistic catalysis; targeted substances (such as ferritin [25]) are assembled to help nanozymes enter more acidic lysosomes aiming to improve catalytic efficiency. However, these synergistic treatment strategies still suffer from various complications. The low stability and combinatory effect ratio of composite materials [26], restricted penetration depth and localized irradiation of the external energy field [27] hamper the actual therapeutic efficiency. The limitations of synergistic treatment and trace amounts of H_2_O_2_ in the TME ask for new POD-mimicking nanozymes with a higher catalytic activity to make the best harness of trace H_2_O_2_ in the TME to enhance tumor treatment efficiency.

Carbon-based POD nanozymes are believed to have a high biocompatibility and tunable enzyme-like activity and offer more opportunities for forthputting in organisms than metallic nanozymes [28]. The catalytic pathway of POD-mimicking activity usually involves •OH generation and electron transfer processes [29]. Based on the catalysis mechanism, graphene, which possesses a large specific surface area, has a greater opportunity over other carbon materials (such as carbon nanotubes and carbon dots) to become a highly active catalyst [30]. Improving the nature and density of active sites on graphene materials can increase the catalytic activity to a certain extent [31]. The study of the mechanisms of oxygen evolution reaction (OER) and oxygen reduction reaction (ORR) has shown that quaternary nitrogen (N) and pyridine N are potential active sites [32]. N-doped graphene is emerging in biomedical applications due to its biocompatibility, redox ability and stability impacted by doping. N-doped graphene exerts a POD-like activity in the acidic environment (around pH = 4) and good biocompatibility is combined with Fe_3_O_4_ on the surface of DNA for catalytic antibacterial treatment [33]. The direct proportional relationship of a POD-like catalytic activity of N-doped graphene with an H_2_O_2_ concentration is used to determine the amount of H_2_O_2_ in a pH = 3~4 acidic environment, thereby detecting biological macromolecules such as glucose whose metabolite is H_2_O_2_ [34]. However, current N-doped graphene displays a POD-like catalytic activity in a low pH acidic environment. For tumor treatment by TME, N-doped graphene is expected to possess an efficient catalytic activity in a weak acidic environment (pH = 5.8~6.5) to be capable of dealing with trace amounts of H_2_O_2_. To date, N-doped graphene has not been reported as having been used in tumor treatment. N doping can introduce a large number of vacancies to graphene thereby increasing the defect density and number of active sites and improving the catalytic activity. In addition, the C/N ratio and the formation of various N functional groups play important roles in the electronic pathway [35]. Therefore, an appropriate regulation of N atom doping in graphene can increase the active sites and optimize the electron transfer process by changing the charge distribution to obtain a higher catalytic activity.

**Scheme 1:**
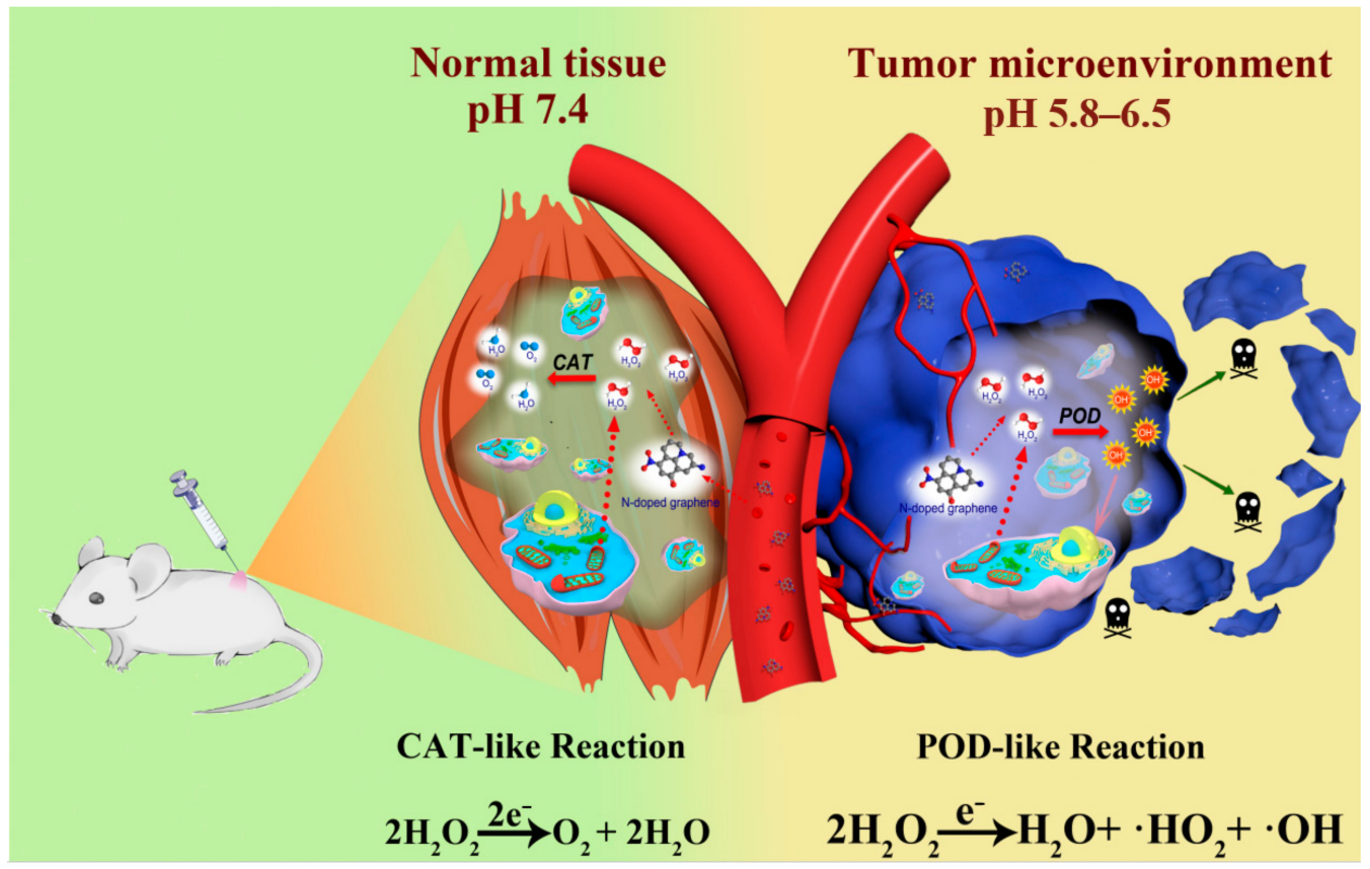
Schematic illustration of the application of N-doped graphene nanomaterials (N-GNMS) for pH-triggered tumor-specific catalytic treatment. Catalase (CAT).

Here, we prepare N-doped graphene nanomaterials (N-GNMs) with high biocatalytic activity for an in vivo tumor-specific catalytic therapy. We confirm that N-GNMs are POD-mimicking nanozymes with a single structure and a high catalytic activity that can respond to endogenous 100 µM–1 mM H_2_O_2_ and weak acids in the TME. Endogenous H_2_O_2_ was used only to produce •OH and cause tumor cell death so as to achieve a sensitive minimum lethal dose antitumor effect. The nanomaterial can be triggered by the weak acidity of the TME. In a weakly acidic environment, it shows the catalytic properties of POD mimics and triggers the processing of the TME but not in other normal tissues with a neutral pH (as shown in Scheme 1). This N-GNM is a non-metallic enzyme with excellent biocompatibility. This is the first application of N-doped graphene materials as catalysts in the biomedical field, providing opportunities for the future application of N-doped graphene and the discovery of new nanozyme materials.

## 2. Materials and Methods

The mouse cervical cancer cell line Hela was obtained from the cell bank of the Chinese Academy of Sciences (Shanghai, China) and the experiments were approved by the Utilization Committee of the South China Normal University (SCNU-BIP-2021-004).

### 2.1. Synthesis of N-Doped Graphene

**Scheme 2:**
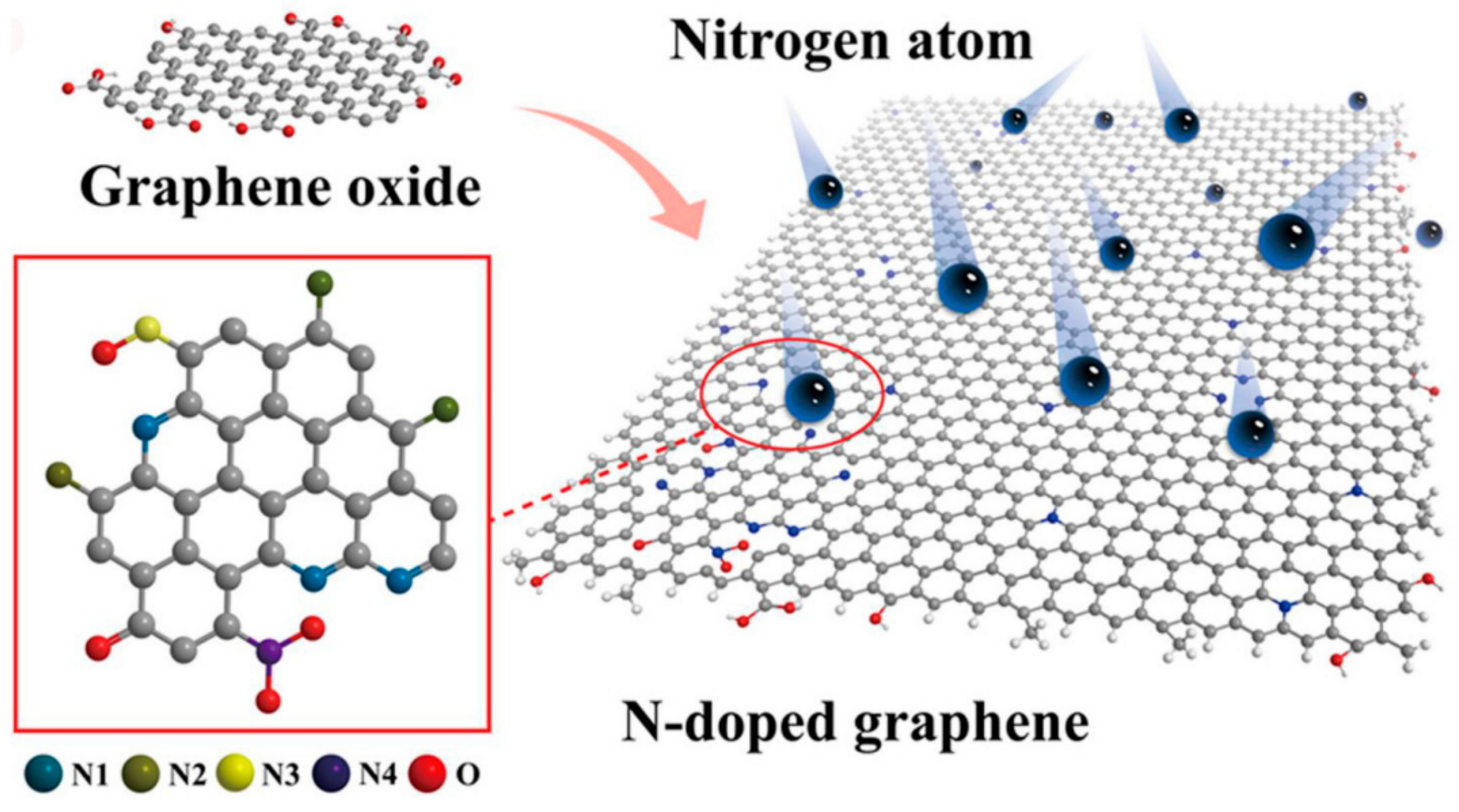
Schematic of the synthesis process of N-GNMs.

The preparation process of N-GNMs is shown in Scheme 2. A total of 7.5 mg graphene oxide (GO) was dissolved into a 15 mL solution. Next, 1 mL of H_2_O_2_ solution with a mass fraction of less than 30% and 0.3 mL of ammonia solution with a mass fraction of less than 28% were added to 15 mL of the GO solution (0.5 mg/mL) and mixed [36]. After uniform mixing, the mixture was transferred to an autoclave lined with Teflon and heated to 180 °C for a constant temperature reaction for 3 h. After cooling to room temperature, the resulting light brown solution was concentrated to one-tenth of its original volume and then dialyzed against ultrapure water (retention molecular weight: 3500 Da) for 48 h.

### 2.2. Characterization of N-Doped Graphene

TEM images were observed with a JEM-2010HR transmission electron microscope (JEOL, Tokyo, Japan). Raman spectra were measured by a Raman spectrometer (Derbyshire, UK). Ultraviolet–visible–near-infrared (UV-vis-NIR) absorption spectra were obtained using an UV-vis-NIR spectrometer (UV-3200S, Mapada, Shanghai, China). Electron spin resonance (ESR) spectra were measured with an ESR spectrometer (Bruker e-scan). XRD was obtained with a Bruker D8 ADVANCE X (Madison, WI, USA). X-ray photoelectron spectroscopy (XPS) spectra were carried out using a Thermo Scientific ESCALAB 250 XI (Waltham, MA, USA). The infrared spectrum was measured with a Fourier transform infrared spectrometer (Nicolet 6700, Thermo Scientific). The particle potential and particle size were determined using a Nano ZS90 dynamic light scattering (DLS) system (Malvern, Surrey, UK). Cell fluorescence imaging was observed using a fluorescence microscope (MSX10, MSHOT, Guangzhou, China).

### 2.3. POD-Like Activity of N-Doped Graphene and Kinetic Assay

A total of 400 μL of 5 mM 3,3′,5,5′-tetramethylbenzidine (TMB) was mixed with different concentrations (0, 0.1, 0.2, 0.3, 0.5, 0.8, 0.9 and 1.0 mg/mL^−1^) of N-GNMs in H_2_O_2_ (1.0 M, 0.8 M, 0.5 M, 0.3 M, 0.1 M, 0.01 M, 1 × 10^−3^ M and 1 × 10^−4^ M) to monitor the chromogenic reaction (λ = 650 nm). A 66.7 mmol.L^−1^ buffer solution of KH_2_PO_4_/Na_2_HPO_4_ was heated at 35 °C for 8 min. One hundred microliters of 0.5 mM terephthalic acid (TA) was mixed with the buffer solution to monitor the fluorescence spectra of the N-GNMs and H_2_O_2_ was excited at a wavelength of 315 nm.

### 2.4. Cell Viability Assay Analysis

The CCK-8 cell viability kit was used to determine the in vitro cytotoxicity of N-GNM. Specifically, HeLa cells were seeded in a 96-well plate at a density of 1 × 10^4^ cells per well and 100 μL of high glucose medium dulbecco’s modified eagle medium (DMEM) containing 10% fetal bovine serum (FBS) were added. The cells were then exposed to a series of N-GNM dilutions prepared with different concentrations of phosphate buffered saline (PBS) and incubated. After incubating for 24 h, 10 μL of CCK-8 solution was added to each well, incubated for 2 h at 37 °C and then the absorbance at 450 nm with the microplate reader was measured.

### 2.5. Cellular Reactive Oxygen Species (ROS) Assay

The fluorescent probe 2′,7′-dichlorofluorescein diacetate (DCFH-DA) was used to observe the generation of ROS. In short, confluent HeLa cells on 6-well plates (2 × 10^5^ cells) were incubated with 0.4 mg/mL^−1^ of N-GNM solution for 48 h. After washing with serum-free DMEM, the cells were incubated in serum-free DMEM with 10 μM DCFH-DA dissolved at 37 °C for 30 min. The fluorescence imaging of DCFH-DA was observed by a fluorescence microscope under a 488 nm excitation and 525 nm emissions. Image J was used to analyze the average green fluorescence intensity.

### 2.6. Intracellular POD Activity

HeLa cells were seeded in 6-well plates (2 × 10^5^ cells), incubated for 12 h and treated with a PBS (0.40 mg/mL^−1^) solution containing N-GNMs for 24 h. Calcein-AM (1 μL) and propidium iodide (PI, 1 μL) were added to the medium to assess cell survival/death, incubated for 15 min, washed with PBS three times and observed through fluorescence microscopy.

### 2.7. Tumor Models

All female BALB/c nude mice were purchased from the Guangdong Medical Laboratory Animal Center and the experiments were approved by the Animal Protection and Utilization Committee of the South China Normal University (SCNU-BIP-2021-004). Approximately 2 × 10^6^ HeLa cells were made into 200 μL PBS cell suspension and injected subcutaneously into the armpit of each mouse to establish the HeLa tumor model. When the tumor volume reached approximately 50 mm^3^ during growth, mice were randomly divided into two groups for different treatments (three mice in each group). Normal saline (200 μL) and N-GMNs (200 μL, 1 mg/mL^−1^) were respectively administered regularly into mouse tumors and they were observed for 14 days. After the mice were sacrificed, the tumor pedicle tissue and main organs were collected for a histological analysis.

## 3. Results and Discussion

### 3.1. Characterization of N-GNMs

**Figure 1:**
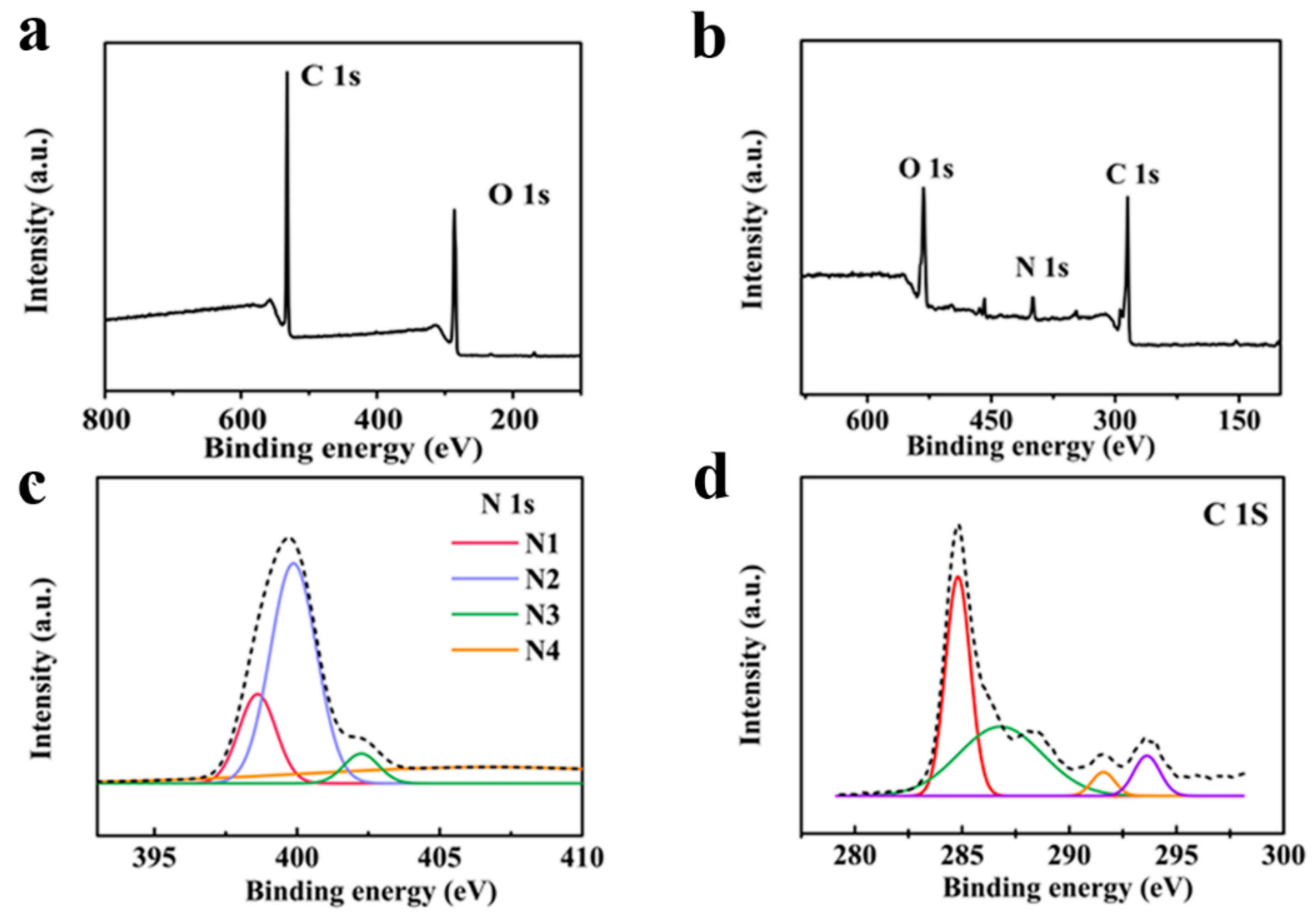
Characteristics of N-GNMs. (**a**) XPS spectrum of the N-GNMs; (**b**) XPS spectrum of the graphene oxide (GO); (**c**) High-resolution XPS spectrum showing the N 1s region of the N-GNMs; (**d**) High-resolution XPS spectrum showing the C 1s region of the N-GNMs.

To characterize the structure of the N-GNMs, we used XPS to analyze their elemental composition. As Figure 1b shows, the scanned spectrum of the N-GNMs showed a C 1s peak (at approximately 283.9 eV) and an O 1s peak (at approximately 531.8 eV). The C/O atomic ratio was approximately 2.47. The N 1s peak was clearly observed in the N-GNMs spectrum at ~399.4 eV and the N/C atomic ratio estimated from the XPS peaks was ~6.17. These elemental analysis results confirmed that N atoms were doped into graphene. At the same time, compared with the XPS spectrum of GO (Figure 1a), a significant decrease in the O content was observed, indicating that the incorporation of nitrogen reduced the oxygen content in the graphene, which might be due to the reduction of graphene by H_2_O_2_ before doping. As Figure 1c shows, the deconvolution of the N 1s peak provided more detailed information about the N functional group. Under different experimental conditions, due to the different doping positions, the N atom could be incorporated into the carbon material to form different kinds of N functional groups such as pyrrolyl, pyridyl, amino and nitrile groups. Therefore, we deconvolved the N 1s peak. The four components were centered at ~398.6, 399.9, 402.5 and 406.7 eV. It is generally considered that the main types of N functional groups are pyridine N (referring to the N atom on the edge of the graphene plane; each atom is bonded to two carbon atoms to provide a pair of lone electrons (N1 in Figure 1c)), pyrrole N (referring to N and two carbon atoms that are bonded and provide two p-electron N atoms (N2 in Figure 1c)), graphitic N (also known as quaternary N or substituted N where N atoms are incorporated into the graphene layer and replace carbon atoms in the plane (N3 in Figure 1c)) and oxidized N (oxidized pyridine dinitrogen, which combines two carbon atoms and one oxygen atom (N4 in Figure 1c)) [32]. The atomic compositions of pyridine, pyrrole, graphite and oxynitride at the surface were ~16.16%, 49.65%, 5.04% and 29.15%, respectively. We speculated that the incorporation of various types of nitrogen atoms could increase graphene defects and active sites.

**Figure 2:**
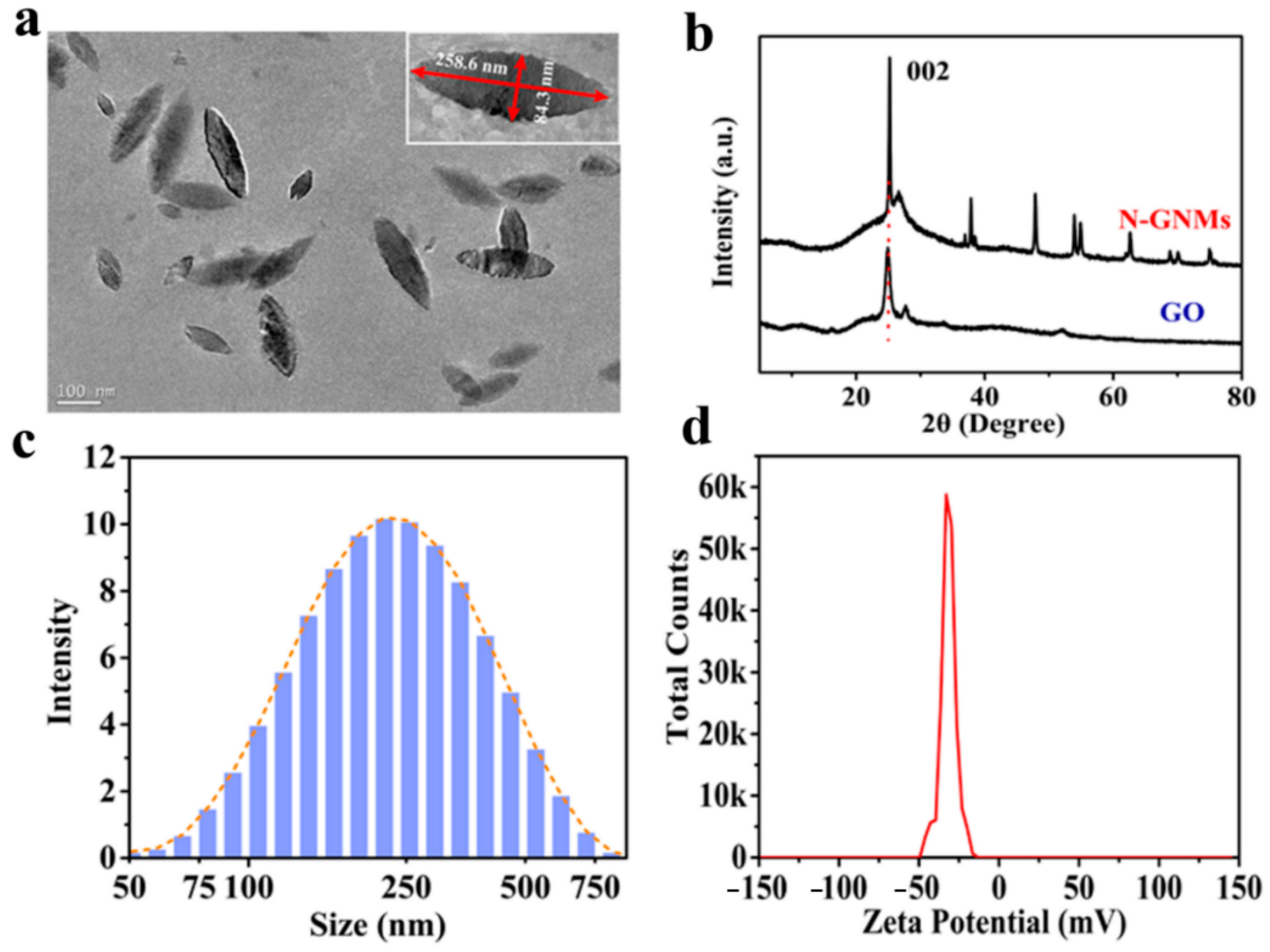
(**a**) TEM images of the N-GNMs; (**b**) XRD patterns of the N-GNMs and GO; (**c**) Particle size distribution and (**d**) Surface potential (ζ) analysis of the N-GNMs.

The TEM images showed that the N-GNMs were nearly fusiform with a uniform size and excellent dispersion. Rod particles with smaller diameters have been shown to have better tumor permeability than spherical particles [37]. As shown in Figure 2a, d = 84.3 nm and h = 258.6 nm. This was fairly consistent with the particle size data measured by DLS and shown in Figure 2c. The aspect ratio was approximately 3.06, which suggested a greater advantage for particle retention in the tumor tissue [38]. The XRD pattern of the N-GNMs (Figure 2b) showed that the diffraction peak at 2θ = 25.5° corresponded with the (002) graphene plane, indicating that the N-GNM material still had the excellent structure of graphene [39]. Figure 2d shows that the zeta potential of the N-GNMs was −32.34 mV and the particle diffusion stability was good.

**Figure 3:**
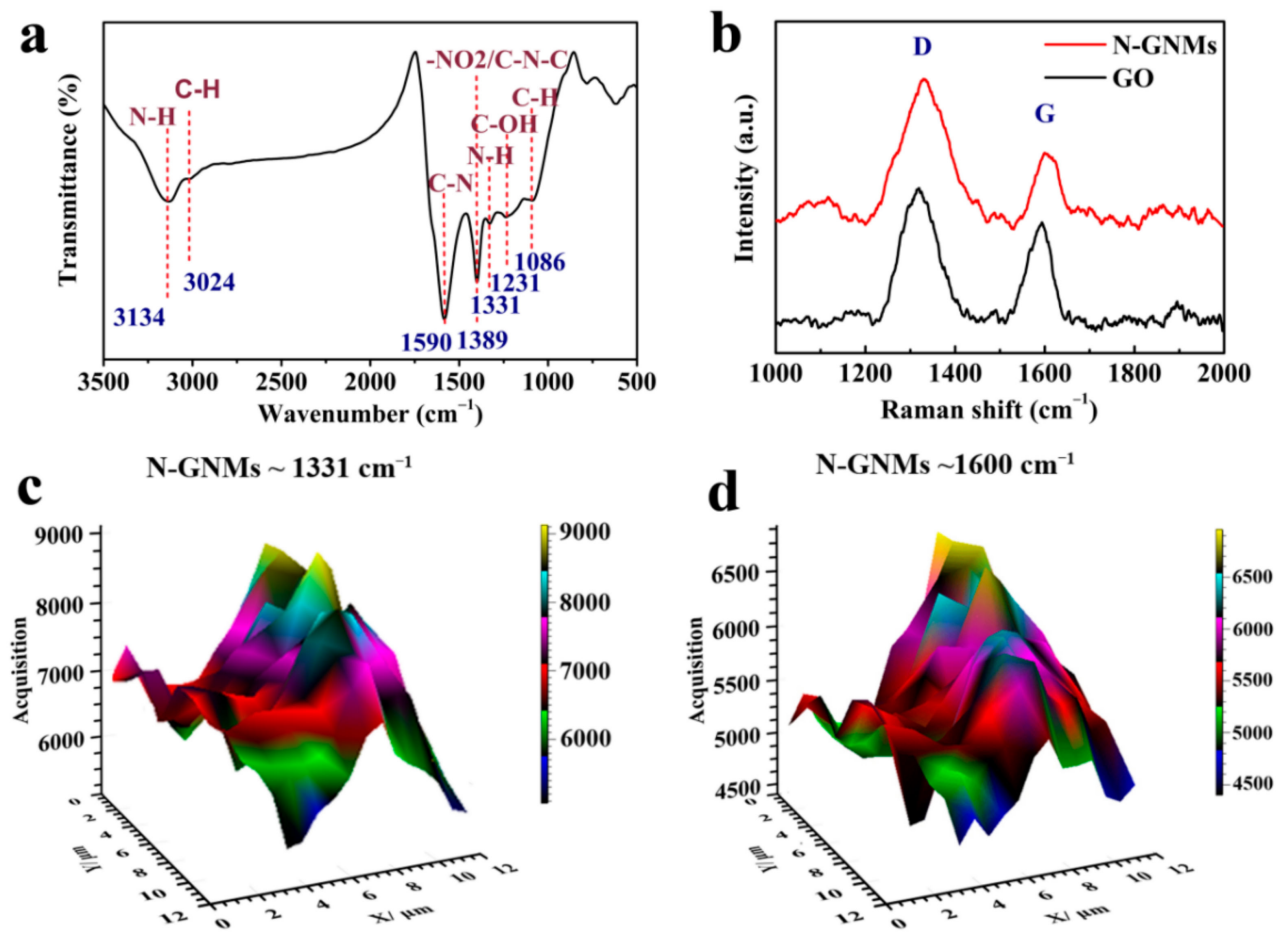
(**a**) Fourier transform infrared (FTIR) spectrum of the N-GNMs; (**b**) Raman spectra of the N-GNMs and GO; 3D spatial Raman mapping for N-GNMs (**c**) at ~1331 cm^−1^ and (**d**) at ~1600 cm^−1^. The field of view was 12 μm × 12 μm with a pixel-to-pixel distance of 2 μm.

In addition, Fourier transform infrared (FTIR) spectra (Figure 3a) were analyzed to determine the surface functional groups of the N-GNMs. Through an FTIR spectroscopy analysis, a few new functional groups were found under a high temperature and high pressure. The stretching vibration of N-H near 3134 cm^−1^ may have been caused by hydrogen bonds. The peaks at 1590 and 1331 cm^−1^ corresponded with the tensile vibrations of C-N and C-N-C, respectively. Furthermore, the typical tensile vibration of C-H at 3024 cm^−1^ was significantly enhanced, indicating that the prepared N-GNMs had a degree of conjugation.

Raman spectroscopy is typically used in chemistry to provide structural fingerprints that can identify different materials and is suitable for characterizing the structure and electronic properties of carbon materials. Figure 3b displays the typical Raman spectra of N-GNMs and GO and two different peaks were observed. After a reduction by H_2_O_2_ and ammonia, the characteristics of the N-GNMs Raman spectrum were similar to those of the GO spectrum (Figure 3b) indicating that the basic structural characteristics of graphene were retained after N doping. However, the intensity of the D band increased indicating that there were more N doping defects. The G peaks for GO and the N-GNMs were present at 1594 and 1600 cm^−1^, respectively. Compared with GO, the downward shift of the G peak in the spectrum of the N-GNMs might be related to the electron donor ability of N heteroatoms. The intensity ratio of the D band to the G band (I_D_/I_G_) used to evaluate graphene defects [40] increased from 1.34 for GO to 1.45 for the N-GNMs, indicating that N doping caused an increase in graphene defects and illustrated that N-GNMs might provide many more chelating sites for small molecules than GO. Figure 3c,d shows 3D Raman maps for GO and the N-GNMs over a 12 μm × 13 μm region at wavelengths of ~1331 and ~1600 cm^−1^, respectively. These maps showed the significant difference in peak intensity, which directly proved that a greater number of defects were present in N-doped graphene than in GO. We confirmed that the experimentally prepared N-GNM material introduced a large number of vacancies into the graphene due to N doping thereby increasing the defect density and the number of active sites. In addition, the carbon to nitrogen ratio and the formation of nitrogen (N) also changed, which changed the material performance from a structural change and improved the catalytic ability.

### 3.2. POD-Like Catalytic Activity of N-GNMs

**Figure 4:**
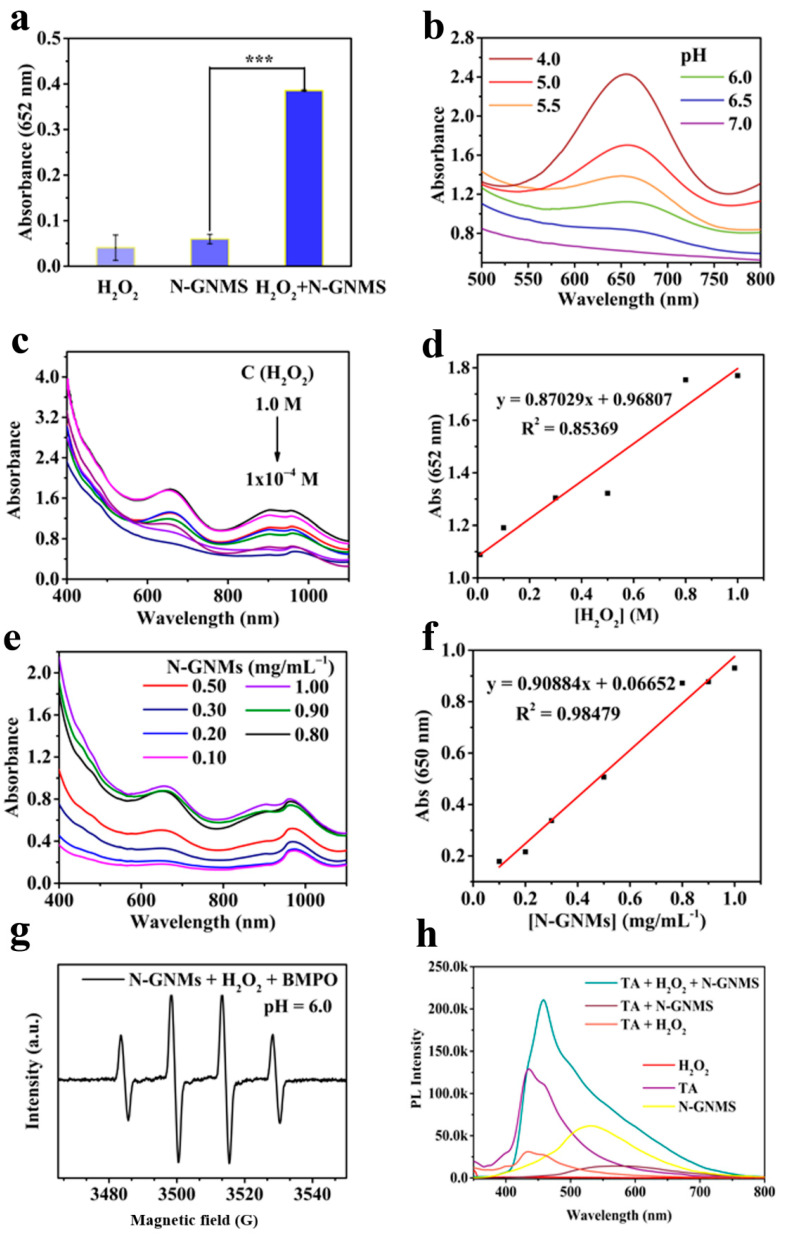
In vitro peroxidase (POD)-like catalytic activity of N-GNMs. (**a**) Catalytic oxidation of 5.0 mM TMB; absorption data for 1.0 M H_2_O_2_, 1 mg/mL^−1^ N-GNMs and 1.0 M H_2_O_2_ with 1 mg/mL^−1^ N-GNMs (*** *p* < 0.001, two-tailed *t* test); (**b**) pH-sensitive POD-like catalytic activity of 1 mg/mL^−1^ N-GNMs; (**c**) Absorbance spectra of the N-GNMs after the addition of different concentrations of H_2_O_2_ (1 × 10^−4^, 1 × 10^−3^, 0.01, 0.10, 0.30, 0.50, 0.80, 1.0 M H_2_O_2_ and 1.0 mg/mL^−1^ N-GNMs). Absorbance spectra of the N-GNMs after the addition of different concentrations of N-GNMs (0.10, 0.20, 0.30, 0.50, 0.80, 0.90, 1.0 mg/mL^−1^ N-GNMs and 1.0 M H_2_O_2_); (**d**) Linear calibration plot for the POD-like catalytic reaction of N-GNMs for H_2_O_2_ detection; (**e**) Absorbance spectra of the N-GNMs after the addition of different concentrations of N-GNMs (0.10, 0.20, 0.30, 0.50, 0.80, 0.90, 1.0 mg/mL^−1^ N-GNMs and 1.0 M H_2_O_2_); (**f**) Linear calibration plot for the N-GNM POD-like catalytic reaction for N-GNM detection; (**g**) Spin-trapped electron spin resonance (ESR) spectrum of •OH radicals in the H_2_O_2_/BMPO system with N-GNMs. (**h**) Fluorescence spectra of the phosphate buffered saline (PBS) solution (pH = 6.0, 10 mM) with TA, H_2_O_2_, N-GNMs, N-GNMs + TA, H_2_O_2_ + TA, TA + N-GNMs + H_2_O_2_.

POD activity is manifested in electron transfer and the generation of •OH. To verify the POD-like activity of the N-GNMs, the TMB colorimetric method was used. The combination of colorless TMB and •OH can produce chromogenic TMB groups and a clear absorption peak can be observed at 650 nm using ultraviolet absorption spectroscopy [35]. As shown in Figure 4a, neither H_2_O_2_ nor N-GNMs alone could oxidize TMB. The combined N-GNMs + H_2_O_2_ + TMB solution was blue and had the characteristic absorption peak of oxidized TMB, indicating that the combination of N-GNMs and H_2_O_2_ could generate •OH. There was a Fenton-like reaction and these results indicated that N-GNMs had a POD-like catalytic activity. As shown in Figure 4b, the absorbance of the TMB color reaction of N-GNMs gradually increased with a decreasing pH. We found that N-GNMs could still exhibit a POD-like activity at pH 6.0, indicating that they could respond to TME in a weakly acidic environment. The continuous increase in the absorbance indicated that the POD-like activity of N-GNMs continued to increase and a POD-like activity was not observed in a neutral environment. This was confirmed by our ESR spectrum at pH = 6.0 (Figure 4g). The absorbance change at the characteristic peak was linearly positively correlated with the concentration of N-GNMs and H_2_O_2_. (Figure 4c–f). The fitting curve for the absorbance and concentration of N-GNMs was A = 0.90884x + 0.06652 mg/mL^−1^ and the correlation coefficient was 0.98479. The fitting curve for the absorbance and H_2_O_2_ concentration was A = 0.87029x + 0.96807 M and the correlation coefficient was 0.85369. N-GNMs that oxidized TMB were affected by the concentration of N-GNMs and H_2_O_2_ and showed a highly linear relationship.

N-GNMs catalyze the disproportionation and decomposition of H_2_O_2_, producing highly toxic •OH under acidic pH conditions and non-toxic O_2_ and H_2_O under neutral pH conditions. Therefore, N-GNMs showed a POD-like activity under acidic conditions and it was inferred that N-GNMs became a POD activity nanozyme in an acidic TME. To observe the ability of N-GNMs to regulate ROS, we measured the in vitro generation of free radicals by electron paramagnetic resonance spectrometry (ESR) [41]. As shown in Figure 4g, a strong characteristic •OH/BMPO peak (1:2:2:1) was observed in the spectrum measured under acidic conditions (pH 6.0), indicating that •OH was produced by the disproportionation of H_2_O_2_ catalyzed by N-GNMs and •OH oxidized TMB to chromogenic TMB under acidic conditions. To further explain the mechanism underlying the POD-like activity of N-GNMs, fluorescence experiments were conducted. As shown in Figure 4h, in the presence of both N-GNMs and H_2_O_2_, the conversion of the non-fluorescent compound TA to the fluorescent product 2-hydroxyterephthalic acid was observed, indicating the formation of •OH radicals [42]. The results of TA fluorescence detection and TMB ultraviolet absorption experiments were consistent. The TA fluorescence detection results were consistent with the results of the TMB ultraviolet absorption experiments, jointly proving that N-GNMs could exert POD activity in a weakly acidic environment and were sensitive to trace amounts of µM H_2_O_2_, reflecting the sensitive weak acid response and high catalytic activity of N-GNMs.

### 3.3. In Vitro Therapeutic Efficacy of N-GNMs

**Figure 5:**
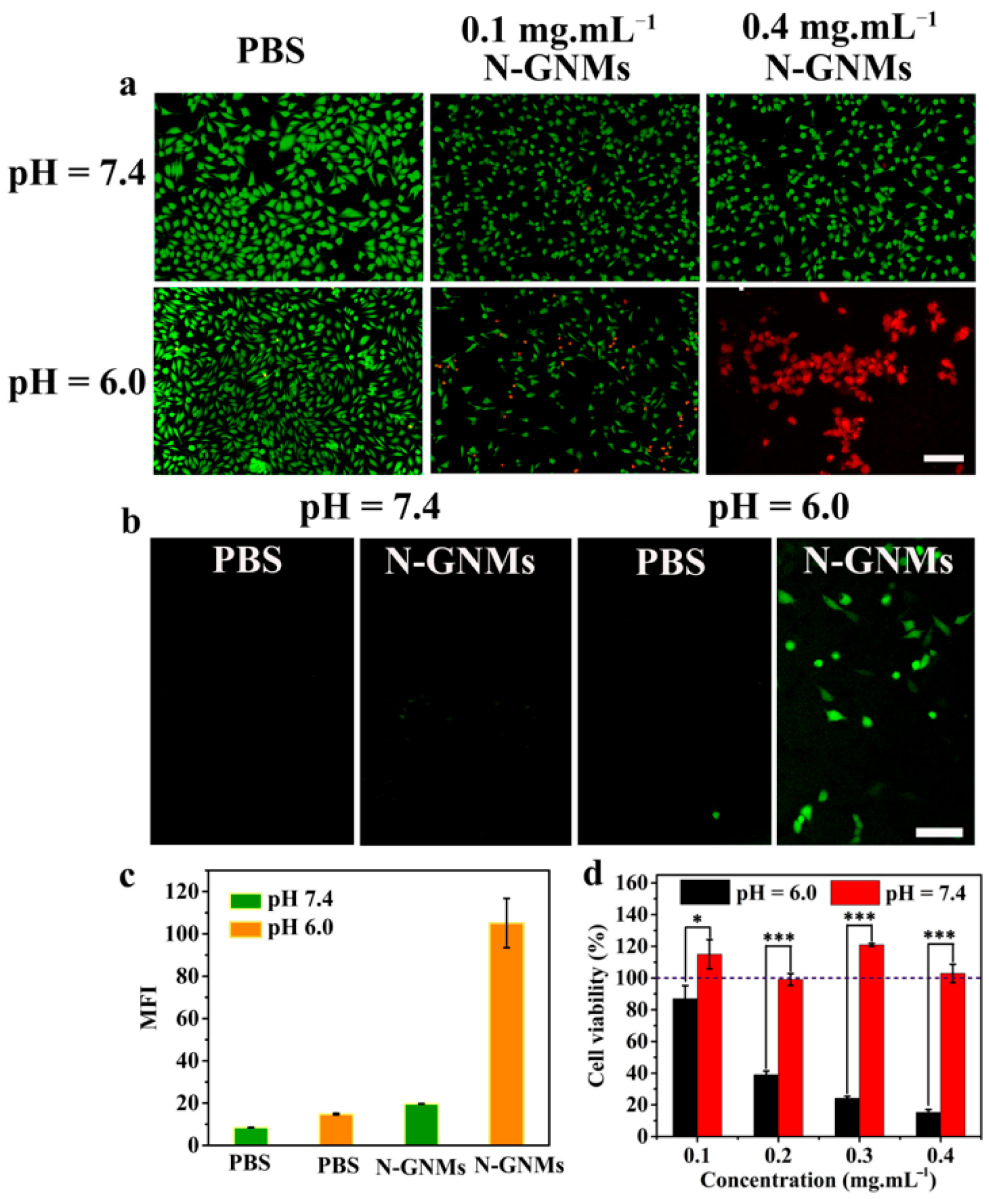
In vitro therapeutic efficacy of N-GNMs. (**a**) Live/dead assay of HeLa tumor cells incubated with 0.40 mg/mL^−1^ N-GNMs under acidic and neutral conditions for 24 h. The cells were stained using calcein-AM/PI. Scale bar: 200 μm; (**b**) HeLa tumor cells incubated with 0.40 mg/mL^−1^ N-GNMs in acidic and neutral media that contained the reactive oxygen species (ROS) fluorescent probe 2′,7′-dichlorofluorescein diacetate (DCFH-DA) for 24 h. Scale bar: 200 μm; (**c**) The mean fluorescence intensity (MFI) of HeLa cells treated with PBS or N-GNMs (0.4 mg/mL^−1^) for 12 h was stained with DCFH; (**d**) Cell viability (as determined by the CCK-8 assay) of HeLa cells treated with N-GNMs under acidic and neutral conditions (* *p* < 0.05, *** *p* < 0.001, two-tailed *t* test).

Encouraged by the efficient •OH production, we used pH 7.4 PBS and pH 6.0 medium solutions to simulate the neutral environment of normal tissues and weakly acidic TME, respectively, without adding any exogenous H_2_O_2_. The cells were incubated with calcein-AM, which exhibited a green fluorescence after excitation and propidium iodide (PI), which exhibited a red fluorescence and the fluorescence images were observed to further confirm the effect of N-GNMs on HeLa cells under different experimental conditions. As shown in Figure 5a, in the medium at pH 7.4 and 6.0, the control group treated with PBS had no obvious cell damage while the cells of the experimental group treated with 0.1 mg/mL^−1^ N-GNMs had only slight damage. When HeLa cells were incubated with 0.4 mg/mL^−1^ N-GNMs in a pH 6.0 medium, a large amount of apoptosis was observed. After incubation with 0.4 mg/mL^−1^ N-GNMs in a pH 7.4 medium, most HeLa cells were still alive. It could be inferred that the catalytic properties of N-GNMs had pH selectivity and no cytotoxicity under neutral conditions but a high toxicity to tumor cells in a weakly acidic environment. In addition, the cytostatic effect of N-GNMs was related to their concentration.

The DCFH-DA fluorescent dye, which is widely used in intracellular ROS probes, was used to prove the mechanism of cell death caused by hydroxyl radicals generated by the catalytic reaction. DCFH is oxidized by ROS and exhibits green fluorescence after being excited by a specific wavelength. As shown in Figure 5b, in a weakly acidic environment (pH = 6.0) that simulates the TME, HeLa cells treated with N-GNMs showed a stronger fluorescence than the control group and it was speculated that a large amount of •OH was produced. As shown in Figure 5c, the mean fluorescence intensity (MFI) of the DCFH staining was analyzed and similar results were obtained. The ROS generated only in the acidic environment confirmed that N-GNMs had sensitive pH-selective POD-mimicking nanozyme activity at the cell level.

To further quantify the catalytic activity of N-GNMS, a CCK-8 kit was used to further analyze the in vitro cytotoxicity of N-GNMs. As shown in Figure 5d, using the above experimental conditions to separately control the pH and N-GNMs concentration, the results showed that N-GNMs exhibited a synergistic dose-dependent and pH-dependent cytotoxicity on cell inhibition. In a pH 7.4 medium, N-GNMs did not affect or even promote cell growth. However, in an acidic (pH 6.0) simulated medium, the TME cells were incubated with N-GNMs and a certain concentration of N-GNMs might cause a significant decrease in cell viability. This showed that N-GNMs could specifically inhibit cancer cells and did not affect normal tissues. The comparison of both N-GNMs and graphene oxide in a tumor treatment showed that the tumor cell lethal rate induced by graphene oxide was 80% when 0.58 mg/mL was applied while the tumor cell lethal rate of N-GNMs was 87% when 0.4 mg/mL was used. As the same concentration (0.3 mg/mL) of graphene oxide and N-GNMs was adopted, the tumor cell lethal rate caused by graphene oxide was about 30% but by N-GNMs it was 75% [35]. Additionally, the IC_50_ of the in vivo N-GNM catalyzed treatment was significantly lower than that of the graphene oxide catalyzed Fenton reaction treatment, indicating that N-GNMs alone could achieve a higher catalytic treatment effect with a lower dosage, which reflected that N doping significantly improved the catalytic activity of graphene oxide materials.

### 3.4. In Vivo Therapeutic Efficacy of N-GNMs

**Figure 6:**
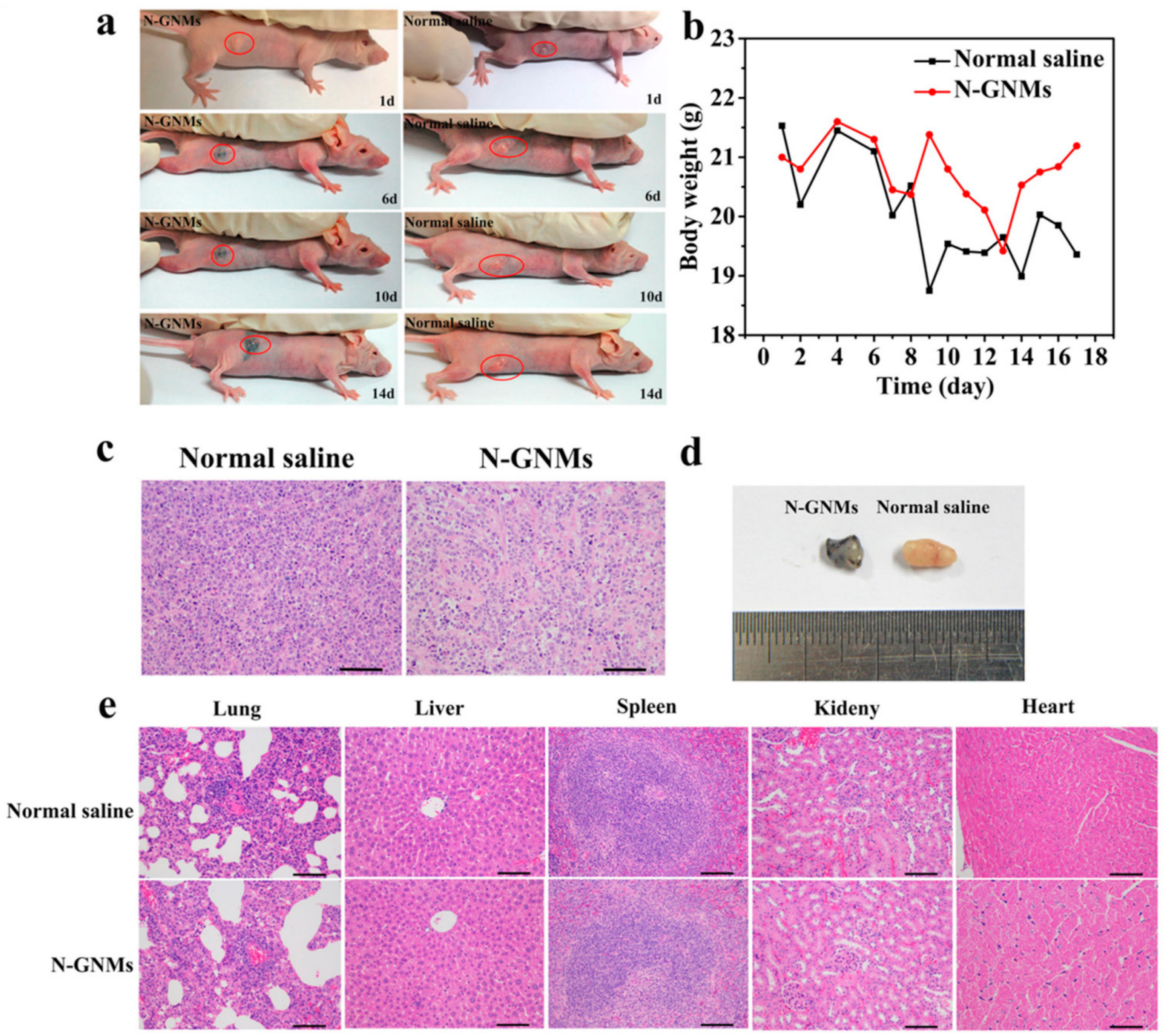
In vivo cancer therapy efficacy. (**a**) Representative photos of mice after different treatments for 1, 6, 10 and 14 days; (**b**) Representative tumor tissues collected from different groups at 14 days; (**c**) Hematoxylin and eosin (H and E) stained slices of tumor tissues from different groups collected at 14 days. Scale bar: 100 μm; (**d**) Body weight changes in HeLa tumor-bearing mice after various treatments for 14 days; (**e**) Histological H and E staining of major organs in nude mice after 14 days. Scale bar: 100 μm.

The previously observed in vitro antitumor efficacy of N-GNMs encouraged us to further investigate its tumor suppressive effect in vivo. HeLa tumor-bearing nude mice (female, four weeks) were used as in vivo tumor models and randomly divided into two groups (n = 3). The tumors were treated with saline and N-GNMs to study the inhibition of tumor growth by the different treatment methods. As shown in Figure 6a,b, normal saline showed no inhibitory effect on the tumor growth. However, tumors in mice treated with N-GNMs showed a significant growth inhibition, which indicated that N-GNMs had an inhibitory effect on the tumor growth. After the mice were sacrificed, the tumor tissues and main organs were stained with hematoxylin and eosin (H and E) to confirm the anticancer effect and biocompatibility of different treatment methods. As shown in Figure 6c, there was a significant difference in the H and E staining between the saline-treated control group and the N-GNM experimental group. In contrast to the normal morphology of the membrane and nucleus of the tumor cells in the saline treatment group, the tumors treated with N-GNMs showed severe membrane morphology and nuclear structure destruction, indicating that N-GNMs could inhibit tumor cell growth through catalytic therapy and the material exhibited highly effective in vivo nanocatalytic therapeutic effects. In addition, the main organs of the mice in the N-GNM injection group and the saline control group were subjected to an H and E staining histological examination. As shown in Figure 6e, there was no significant difference in all groups and the body weight of the mice did not change significantly during the observation experiment (Figure 5d), demonstrating that the N-GNMs showed a low toxicity in these animals. In the acidic TME, N-GNMs exhibited mimicked POD nanozyme activity, decomposed endogenous H_2_O_2_ in tumors and inhibited tumor growth. In the non-tumor areas, the neutral environment did not produce toxic •OH. Due to the high biocompatibility and high catalytic activity of N-GNMs, a proper amount of nanozyme materials is not toxic to animals. To further evaluate the effect of N-GNMs on mouse systemic circulation, N-GNMs and normal saline were injected intravenously into healthy female BALB/c mice to monitor the changes in white blood cell (WBC), red blood cell (RBC), hemoglobin(HGB), hypersensitive C-reactive protein (HS-CPR) and red blood cell distribution width (RDW) values. For the purpose of determining whether the immune system was abnormal, a routine blood analysis was performed three days after the injection of the drug and no obvious abnormality was found (*p* < 0.05). The results showed the high biocompatibility of N-GNMs (Table 1).

## 4. Conclusions

In summary, our research has found that N-doped graphene nanomaterials (N-GNMs) were a nanozyme with efficient POD-like biocatalytic performance for tumor-specific treatment. Furthermore, we proved that N-GNMs had a high catalytic activity, sensitive pH selectivity and trace endogenousH_2_O_2_ concentration (100 µM–1 mM) responsiveness, which could be used in the TME. At a weakly acidic pH, N-GNMs catalyzed decomposition of the low toxicity reactive oxygen species H_2_O_2_ into highly toxic •OH. Under neutral pH conditions of normal tissue, N-GNMs split H_2_O_2_ into non-toxic H_2_O and O_2_. Combining the pH selectivity of this nanozyme with the unique physical and chemical properties of the TME, this could act as a new tumor-specific treatment therapy. In addition, our highly catalytically active POD-mimicking nanozyme N-GNMs responded to the trace amount of endogenous H_2_O_2_ in the TME for efficient tumor treatment. This specific method of improving the biological activity of materials was designed to provide ideas for the development of tumor catalytic therapy drugs with significant curative effects. This is the first time that N-GNMs have been found to have a biocatalytic function. Furthermore, this is the first time that they have been used in the body as a tumor treatment. As a new type of POD-mimicking nanozyme first applied in tumor therapy, N-GNM expands the family of nanozymes and broadens the applications of N-doped graphene materials in biomedicine as well.

## Figures and Tables

**Table 1:** Determination of the blood components of BALB/c Mice.

Index	N-GNMs	Normal Saline
WBC (10^9^/L)	4.57 ± 1.22	3.79 ± 0.32
RBC (10^11^/L)	9.51 ± 0.22	9.22 ± 0.15
RBC (10^11^/L)	141 ± 11.00	141 ± 0.00
RDW-SD (%)	18.3 ± 4.50	16.8 ± 0.40
HS-CPR (mg/L)	0.06 ± 0.06	0.00 ± 0.00

WBC: leukocyte; RBC: red blood cell; HGB: hemoglobin; RDW-SD: coefficient of variation in RBC distribution width; HS-CPR: high sensitivity C-reactive protein. Statistical analyses showed that there were no significant differences between the N-GNM and normal saline groups (*p* < 0.05, *n* = 4).

## Data Availability

Data are contained within the article.
